# Pathway-based analysis of genome-wide association study of circadian phenotypes

**DOI:** 10.7555/JBR.32.20170102

**Published:** 2018-02-19

**Authors:** Di-di Zhu, Jia-min Yuan, Rui Zhu, Yao Wang, Zhi-yong Qian, Jian-gang Zou

**Affiliations:** 1. Department of Cardiology, the First Affiliated Hospital of Nanjing Medical University, Nanjing, Jiangsu 210029, China; 2. Department of Cardiology, the First Affiliated Hospital of Soochow University, Suzhou, Jiangsu 215006, China.

**Keywords:** circadian phenotypes, genome-wide association studies, pathway-based analysis

## Abstract

Sleepiness affects normal social life, which attracts more and more attention. Circadian phenotypes contribute to obvious individual differences in susceptibility to sleepiness. We aimed to identify candidate single nucleotide polymorphisms (SNPs) which may cause circadian phenotypes, elucidate the potential mechanisms, and generate corresponding SNP-gene-pathways. A genome-wide association studies (GWAS) dataset of circadian phenotypes was utilized in the study. Then, the Identify Candidate Causal SNPs and Pathways analysis was employed to the GWAS dataset after quality control filters. Furthermore, genotype-phenotype association analysis was performed with HapMap database. Four SNPs in three different genes were determined to correlate with usual weekday bedtime, totally providing seven hypothetical mechanisms. Eleven SNPs in six genes were identified to correlate with usual weekday sleep duration, which provided six hypothetical pathways. Our results demonstrated that fifteen candidate SNPs in eight genes played vital roles in six hypothetical pathways implicated in usual weekday bedtime and six potential pathways involved in usual weekday sleep duration.

## Introduction

Sleepiness impairs social function, reduces quality of life and causes occupational and motor vehicle accidents^[1]^. While behavioral factors, circadian factors (time of day), duration of wakefulness and sleep disorders are closely linked to daytime sleepiness^[2]^, there are great interindividual differences in susceptibility to sleepiness^[3]^. Accumulating evidence shows that excessive sleepiness is heritable^[4–5]^. In modern society, nearly one-fifth of employees are involved in long-term night shift^[6]^. As a result, work performance and scheduling have a significant impact on individual variability in diurnal preference. Studies also indicate that diurnal preference (namely usual weekday bedtime) is heritable^[7–9]^. In addition, usual weekday sleep duration plays a critical role in daytime sleepiness. It has been investigated whether short or long sleep duration has been related to coronary heart disease^[10]^, diabetes mellitus^[11–12]^, hypertension^[13]^, and mortality^[14]^. Likewise, usual day sleep duration is heritable^[15]^.

To date, several single nucleotide polymorphisms (SNPs) associated with circadian phenotypes in some genes were detected from three genome-wide association studies (GWASs)^[16–18]^, but the functions of these SNPs remain undefined, which is a challenge in interpreting GWAS results^[19]^. Thus, pathway-based approaches were optimized gradually, and the Identify Candidate Causal SNPs and Pathways (ICSNPathway) was created to determine potential SNPs and hypothetical mechanisms through GWAS data, using linkage disequilibrium (LD) analysis, functional SNP annotation and pathway-based analysis (PBA)^[20]^. Herein, we used bioinformatics methods combining ICSNPathway analysis and HapMap database to identify candidate SNPs and relevant pathways, aiming to develop SNP-gene-pathway hypotheses regarding circadian phenotypes.

## Materials and methods

### Study population and data extraction

We applied publicly available databases to identify eligible GWASs on circadian phenotypes, which are the National Human Genome Research Institute GWAS catalog (http://www.genome.gov/26525384), the National Center for Biotechnology Information (NCBI) dbGap (http://www.ncbi.nlm.nih.gov/gap/), and the GWAS central (http://www.gwascentral.org/). In addition, both EMBASE and PUBMED databases were searched with the following key words: “GWAS” or “genome-wide association study” and “circadian”. All searches were completed up to April 20th, 2016 without language limitation. In order to reduce the effect of genotyping errors, two independent authors (DZ and JYuan) filtered the primary GWAS data set and removed individuals with a call rate<95%, minor allele frequency<0.01, and deviating from the Hardy-Weinberg equilibrium (HWE) test (*P*<0.001). During data extraction, discussion with a third author (YW) helped resolve the discrepancies, with consensus on each item reached in the end. After extracting data from the original papers and contacting the corresponding authors, we ruled out the studies without details as needed.

### Identification of candidate causal SNPs and pathways

ICSNPathway analysis was conducted in two consecutive stages. In the first stage, the candidate SNPs were pre-selected by LD analysis and functional SNP annotation with *P* values of <0.05^[20]^. During the LD analysis, we queried GWAS to capture the SNPs in LD (with *r*^2^>0.8) and positioned in the flanking region (with up to 500 kb upstream and downstream) . The extended dataset including HapMap data (http://hapmap.ncbi.nlm.nih.gov) was utilized to obtain more possible candidate SNPs^[21]^. Additionally, to gain LD structures, we used SNAP dataset (http://www.broadinstitute.org/mpg/snap/)^[22]^. The other method involves the functional annotation on the SNPs by searching the international SNP function annotation databases, including PolyPhen-2 (http://genetics.bwh.harvard.edu/pph2/)^[23]^, Ensembl database (http://www.ensembl.org)^[24]^, SNPs3D (http://www.snps3d.org)^[25]^, and SIFT (http://sift.jcvi.org)^[26]^.

Genotypic frequencies of candidate SNPs was extracted from the International HapMap Project (phase II, release 23), consisting of 3.96 million SNP genotypes from 270 subjects^[27]^. Besides, the data of corresponding mRNA expression was acquired from lymphoblastic cell lines of the 270 individuals mentioned above^[28]^, which was extracted from SNPexp (http://app3.titan.uio.no/biotools/help.php?app=snpexp/)^[29]^.

During the second stage, PBA algorithm was employed to annotate biological pathways of selected SNPs by integrating data from four databases, including BioCarta (http://www.biocarta.com), MsiDB (http://www.broadinstitute.org/gsea/msigdb), Kyoto Encyclopedia of Genes and Genomes (KEGG, http://www.genome.jp/kegg) and gene ontology (GO, http://www.geneontology.org). Furthermore, SNP label normalization and permutation were adopted to correct gene variations and generate the distribution of significant proportion based enrichment score (SPES).According to the distributions of SPESs, a nominal *P*-value and a FDR (false discovery rate; cutoff value: 0.05) were calculated.

### Statistical analysis

The expression levels were shown as mean±SEM, and the difference between two genotypes was evaluated by two-side Student's *t* test. Furthermore, one way ANOVA was utilized to assess the difference of transcript expression levels in more than two genotypes. The statistical analysis was performed with SPSS version 21.0. *P* values<0.05 were considered statistically significant.

## Results

### Characteristics of the study population

One GWAS drawn from NCBI dbGap (study accession: phs000007) was finally adopted in our study^[16]^ with publicly available summary data after a thorough search. In the GWAS on circadian phenotypes(including usual weekday bedtime and usual weekday sleep duration), totally 749 subjects were collected from the Framingham Offspring Study containing 2848 participants who accomplished sleep habit questionnaires between 1995 and 1998 (Offspring Examination Cycle 6) for the Sleep Heart Health Study^[30]^. For usual weekday bedtime, 65,514 candidate causative SNPs were originally generated with an Affymetrix 100K SNP Gene Chip, and afterwards 47,285 SNPs passed the quality control filters which were employed for ultimate bioinformatics analysis. Besides, for usual weekday sleep duration, 65,514 SNPs were generated with the gene chip, while 47,301 SNPs met the quality control criterions and were then applied for subsequent analysis.

### Candidate SNPs and pathways

**Fig.1:**
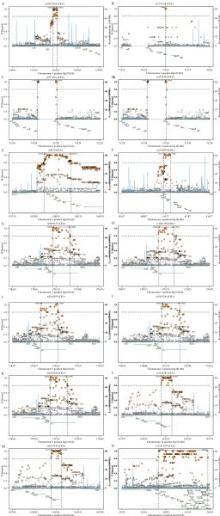
Detailed LD plots for the polymorphisms.

As presented in ***Table 1***, totally four SNPs in three genes were determined to correlate with usual weekday bedtime, namely, MT-ND5 rs10517616, GRSF1 rs3775728, and ENAM rs7671281, rs3796704 polymorphisms. Moreover, eleven SNPs in six genes were identified to correlate with usual weekday sleep duration, namely, HSPD1 rs8539, APOBEC2 rs2076472, GRSF1 rs3775728, TTN rs9808377, rs1001238, rs2042995, rs3829746, rs2042996, CENPE rs2243682, rs2615542 and SLC17A1 rs13213957. Of note, GRSF1 rs3775728 was linked with both usual weekday bedtime and usual weekday sleep duration. SNP rs3775728 was in LD with rs2278134 (*r*^2^=1.0) ; rs7671281 and rs3796704 were in LD with rs2553319 (*r*^2^=1.0, and 1.0, respectively); rs9808377, rs1001238 and rs2042995 were in LD with rs3829746 (*r*^2^=0.945, 0.946, and 0.945, respectively); rs2243682 and rs2615542 were in LD with rs2290943 (*r*^2^=1.0, and 1.0, respectively); SNP rs13213957 was in LD with rs3734523 (*r*^2^=0.828). Except for a repeated SNP, fourteen regional LD plots are shown in ***Fig. 1***.

**Tab.1:** Candidate single nucleotide polymorphisms identified by ICSNPathway analysis

SNP ID	Functional class	Gene	Chromosome	Candidate- pathway^a^	-log_10_(P)^b^	In LD with	*r*^2^	D'	-log_10_(P)^c^
Usual weekday bedtime									
rs10517616	nonsynonymous coding	*MT-ND5*	4	1,2,4,6	1.49	rs10517616	NA	NA	1.49
rs3775728	nonsynonymous coding	*GRSF1*	4q13	3	NA	rs2278134	1	1	1.664
rs7671281	nonsynonymous coding	*ENAM*	4q13.3	5	NA	rs2553319	1	1	1.342
rs3796704	nonsynonymous coding(deleterious)	*ENAM*	4q13.3	5	NA	rs2553319	1	1	1.342
Usual weekday sleep duration									
rs8539	nonsynonymous coding	*HSPD1*	2q33.1	1	1.62	rs8539	NA	NA	1.62
rs2076472	nonsynonymous coding	*APOBEC2*	6p21	2	1.533	rs2076472	NA	NA	1.533
rs3775728	nonsynonymous coding	*GRSF1*	4q13	2,4,6	NA	rs2278134	1	1	1.881
rs9808377	nonsynonymous coding	*TTN*	2q31	3	NA	rs3829746	0.945	1	1.567
rs1001238	nonsynonymous coding	*TTN*	2q31	3	NA	rs3829746	0.946	1	1.567
rs2042995	nonsynonymous coding	*TTN*	2q31	3	NA	rs3829746	0.945	1	1.567
rs3829746	nonsynonymous coding	*TTN*	2q31	3	1.567	rs3829746	NA	NA	1.567
rs2042996	nonsynonymous coding	*TTN*	2q31	3	1.423	rs2042996	NA	NA	1.423
rs2243682	nonsynonymous coding(deleterious)	*CENPE*	4q24-q25	3	NA	rs2290943	1	1	1.418
rs2615542	nonsynonymous coding	*CENPE*	4q24-q25	3	NA	rs2290943	1	1	1.418
rs13213957	regulatory region	*SLC17A1*	6p22.2	5	NA	rs3734523	0.828	1	1.605

SNP: single nucleotide polymorphism; LD: linkage disequilibrium; NA: not available. ^a ^The number indicates the index of pathways ranked by their statistical significance (false discovery rate). ^b^−log_10_(P) for candidate SNP in the original genome wide association study (GWAS). ^c ^−log_10_(P) for the SNP in the original GWAS which was in LD with candidate SNP.

Then, we examined the roles of different genotypes in mRNA expression levels via HapMap c-DNA expression database which was publicly available. No significant association between all SNPs with the mRNA expressions of corresponding genes was found in Caucasians as presented in ***Table 2***. However, the SLC17A1 rs13213957 polymorphisms might tend to affect the mRNA expression levels of SLC17A1 (with marginal *P* value=0.0785), which is consistent with the functional class indicated in ***Table 1***. In addition, the functions of the corresponding proteins were examined, which demonstrated that all SNPs could cause residue change except for HSPD1 rs8539, summarized in ***Table 3***. In addition, MT-ND5 rs10517616 was not estimated here because no data was available publicly.

**Tab.2:** mRNA expression by the genotypes of SNPs with the data from HapMap

Category	No.	Mean±SEM	*P*^a^	*P*^b^
rs3775728				
TT	55	10.56±0.04378		NA
CT	3	10.84±0.11230	0.1536	
rs7671281				
TT	52	6.067±0.01329		NA
CT	4	6.046±0.03729	0.6837	
rs3796704				
GG	53	6.065±0.01309		NA
AG	3	6.062±0.04807	0.9500	
rs8539				
CC	31	6.198±0.01135		0.9718
CT	19	6.190±0.01628	0.6832	
TT	5	6.194±0.02530	0.8887	
CT+TT	24	6.191±0.01370	0.6868	
rs2076472				
TT	37	6.311±0.01794		NA
CT	19	6.291±0.01970	0.4927	
rs9808377				
AA	37	6.426±0.01331		0.7668
AG	16	6.409±0.01801	0.4692	
GG	3	6.398±0.05742	0.5726	
AG+GG	19	6.408±0.01692	0.3997	
rs1001238				
TT	35	6.427±0.01354		0.7663
CT	18	6.410±0.01765	0.4561	
CC	3	6.398±0.05742	0.5616	
CT+CC	21	6.408±0.01658	0.3918	
rs2042995				
TT	37	6.426±0.01331		0.7668
CT	16	6.409±0.01801	0.4692	
CC	3	6.398±0.05742	0.5726	
CT+CC	19	6.408±0.01692	0.3997	
rs3829746				
TT	36	6.429±0.01335		0.5472
CT	17	6.404±0.01768	0.2779	
CC	3	6.398±0.05742	0.5305	
CT+CC	20	6.403±0.01661	0.2376	
rs2042996				
GG	36	6.426±0.01368		0.787
AG	17	6.410±0.01695	0.4921	
AA	3	6.398±0.05742	0.5787	
AG+AA	20	6.409±0.01608	0.4213	
rs2243682				
GG	38	8.850±0.06324		0.5172
AG	16	8.949±0.11200	0.4196	
AA	1	9.41940207	NA	
AG+AA	17	8.976±0.10870	0.2934	
rs2615542				
AA	38	8.850±0.06324		0.522
AG	17	8.945±0.10520	0.4215	
GG	1	9.41940207	NA	
AG+GG	18	8.972±0.10260	0.2979	
rs13213957				
TT	72	6.067±0.01000		0.1051
CT	16	6.033±0.01920	0.1453	
CC	1	5.926081	NA	
TT+CT	17	6.027±0.01910	0.0785^c^	

SNP: single nucleotide polymorphism; NA: not available. ^a^ Two-side Student's t test within the stratum. ^b ^*P* values for one way ANOVA of mRNA expression among different genotypes for each SNP. ^c^ Marginal *P* value (in bold).

**Tab.3:** Residue changes by the genotypes of SNPs with the data from dbSNP

SNP	Gene	Protein position	Residue change
rs3775728	*GRSF1*	194	Val-to-Ile
rs7671281	*ENAM*	648	Ile-to-Thr
rs3796704	*ENAM*	763	Arg-to-Gln
rs8539	*HSPD1*	91	Lys-to-Lys
rs2076472	*APOBEC2*	136	Ile-to-Thr
rs9808377	*TTN*	20346	Ile-to-Thr
rs1001238	*TTN*	9651	Asn-to-Asp
rs2042995	*TTN*	10221	Ile-to-Val
rs3829746	*TTN*	18725	Ile-to-Val
rs2042996	*TTN*	12353	Thr-to-Ile
rs2243682	*CENPE*	1942	Thr-to-Met
rs2615542	*CENPE*	1535	Phe-to-Leu

SNP: single nucleotide polymorphism.

During the ICSNPathway analysis, six pathways about usual weekday bedtime were detected and are summarized in ***Table 4***. The first mechanism involved MT-ND5 rs10517616 polymorphism (nonsynonymous coding) in pathways such as NADH dehydrogenase activity (nominal *P*<0.001, FDR=0.011), respiratory electron transport chain (nominal *P*=0.001, FDR=0.011), oxidoreductase activity (nominal *P*=0.002, FDR=0.017), and oxidative phosphorylation (nominal *P*=0.004, FDR=0.047). The second was GRSF1 rs3775728 polymorphism (nonsynonymous coding) in mRNA binding pathway (nominal *P*<0.001, FDR=0.014). The third one included ENAM rs7671281, rs3796704 polymorphisms (nonsynonymous coding) in pathway of biomineral formation (nominal *P*<0.001, FDR=0.021).

**Tab.4:** Candidate pathways for circadian phenotypes

Index	Candidate pathway	Description	*P*_N_	FDR
Usual weekday bedtime				
1	NADH dehydrogenase activity	GO:0003954. Catalysis of the reaction: NADH+ H+ + acceptor=NAD+ + reduced acceptor.	<0.001	0.011
2	Respiratory electron transport chain	GO:0022904. A process whereby a series of electron carriers operate together to transfer electrons from donors such as NADH and FADH2 to any of several different terminal electron acceptors to generate a transmembrane electrochemical gradient.	0.001	0.011
3	mRNA binding	GO:0003729. Interacting selectively with pre-messenger RNA (pre-mRNA) or messenger RNA (mRNA).	<0.001	0.014
4	Oxidoreductase activity	GO:0016655. Catalysis of an oxidation-reduction (redox) reaction in which NADH or NADPH acts as a hydrogen or electron donor and reduces a quinone or a similar acceptor molecule.	0.002	0.017
5	Biomineral formation	GO:0031214. Formation of hard tissues that consist mainly of inorganic compounds, and also contain a small amounts of organic matrices that are believed to play important roles in their formation.	<0.001	0.021
6	Oxidative phosphorylation	GO:0006119. The phosphorylation of ADP to ATP that accompanies the oxidation of a metabolite through the operation of the respiratory chain. Oxidation of compounds establishes a proton gradient across the membrane, providing the energy for ATP synthesis.	0.004	0.047
Usual weekday sleep duration				
1	Unfolded protein binding	GO:0051082. Interacting selectively with an unfolded protein.	0.001	0.03
2	mRNA processing	GO:0006397. Any process involved in the conversion of a primary mRNA transcript into one or more mature mRNA(s) prior to translation into polypeptide.	<0.001	0.031
3	Cell cycle	GO:0007049. The progression of biochemical and morphological phases and events that occur in a cell during successive cell replication or nuclear replication events. Canonically, the cell cycle comprises the replication and segregation of genetic material followed by the division of the cell, but in endocycles or syncytial cells nuclear replication or nuclear division may not be followed by cell division.	<0.001	0.036
4	RNA processing	GO:0006396. Any process involved in the conversion of one or more primary RNA transcripts into one or more mature RNA molecules.	0.002	0.039
5	Anion transport	GO:0006820. The directed movement of anions, atoms or small molecules with a net negative charge, into, out of, within or between cells.	<0.001	0.042
6	mRNA binding	GO:0003729. Interacting selectively with pre-messenger RNA (pre-mRNA) or messenger RNA (mRNA).	<0.001	0.042

*P*_N_: nominal *P* value; FDR: false discovery rate; GO: gene ontology.

In the ICSNPathway analysis of usual weekday sleep duration, six pathways were found and are presented in ***Table 4*** similarly. The first was HSPD1 rs8539 polymorphism (nonsynonymous coding) in the unfolded protein binding pathway (nominal *P*=0.001 FDR=0.03). The second one was APOBEC2 rs2076472 polymorphism (nonsynonymous coding) in pathway of mRNA processing (nominal *P*<0.001, FDR=0.031). The third mechanism involved GRSF1 rs3775728 polymorphism (nonsynonymous coding) in pathways containing mRNA processing (nominal *P*<0.001, FDR=0.031), RNA processing (nominal *P*=0.002, FDR=0.039), and mRNA binding (nominal *P*<0.001, FDR=0.042). The fourth pathway consisted of TTN rs9808377, rs1001238, rs2042995, rs3829746, rs2042996, and CENPE rs2243682, rs2615542 polymorphisms (nonsynonymous coding) in cell cycle (nominal *P*<0.001, FDR=0.036). The last one was SLC17A1 rs13213957 polymorphism (regulatory region) in the anion transport pathway (nominal *P*<0.001, FDR=0.042).

## Discussion

A compound molecular network may make a significant contribution to the development of circadian phenotypes, containing several cellular pathways^[31]^. GWASs are limited to detect single SNP associations and identify new loci, so we applied a pathway-based pattern to take the biological interplay between multiple genes into consideration, and propose novel views into how genes might help the development of circadian phenotypes^[32]^.

In this study, we applied ICSNPathway analysis to identify six potential regulating mechanisms, respectively, in usual weekday bedtime and sleep duration. The most significant SNP-to-gene-to-effect hypothesis was that rs10517616 changes the feature of MT-ND5 in NADH dehydrogenase activity^[33]^. It was reported that NADH promoted the transcription of the lactate dehydrogenase (*LDH*) gene under redox state. This is based on the activation of *E*-box by binding heterodimer Bmal1/NPAS2, the master brain clock to regulate circadian rhythmicity^[^34a^]^. The second candidate gene GRSF1 found in this study and previous studies has been implied in the pathway of mRNA binding through SNP rs3775728^[34–35]^. The third biological mechanism involves the modulation of ENAM by rs7671281 and rs3796704 to affect its role in mineral formation^[36–37]^. The forth one involves the influence of rs8539 on HSPD1 in unfolded protein binding^[38]^. The fifth involves the modulation of APOBEC2 by rs2076472 to affect mRNA processing. The sixth involves the modulation of TTN by rs9808377, rs1001238, rs2042995, rs3829746, and rs2042996 as well as CENPE by rs2243682 and rs2615542 to influence its role in cell cycle^[39]^. The seventh involves the modulation of SLC17A1 by rs13213957 to affect anion transport^[40–41]^, which could influence the mRNA expression of SLC17A1.

As far as we know, these mechanisms of circadian phenotypes, including MT-ND5, GRSF1, ENAM, HSPD1, APOBEC2, TTN, CENPE and SLC17A1, have been firstly identified in our study. The ICSNPathway analysis has been conducted to identify candidate causal genes relevant to disease-related phenotypes such as rheumatoid arthritis^[20]^. Thus, the results received in our study might help the development of novel hypotheses for the further investigations.

Even though the abovementioned biological mechanisms may affect circadian phenotypes, several limitations should be acknowledged. Firstly, the data was obtained from only 749 subjects^[16]^, which may limit the application to the whole populations and weaken the authority to identify the candidate SNPs. Secondly, with no study supplying strong supports for these results, the candidate SNP-gene-pathways should be verified in more studies.

In short, our results demonstrated fifteen candidate SNPs in eight genes (MT-ND5 rs10517616, GRSF1 rs3775728, ENAM rs7671281, rs3796704, HSPD1 rs8539, APOBEC2 rs2076472, GRSF1 rs3775728, TTN rs9808377, rs1001238, rs2042995, rs3829746, rs2042996, CENPE rs2243682, rs2615542 and SLC17A1 rs13213957 polymorphisms), which participate in six hypothetical pathways involved in usual weekday bedtime and six potential pathways implicated usual weekday sleep duration. However, further investigations are warranted to validate the identified genetic variations in the biological pathways related to circadian phenotypes.
